# Overexpression of peptidase inhibitor 16 attenuates angiotensin II–induced cardiac fibrosis via regulating HDAC1 of cardiac fibroblasts

**DOI:** 10.1111/jcmm.15178

**Published:** 2020-03-30

**Authors:** Mengqing Deng, Shuo Yang, Yue Ji, Yan Lu, Ming Qiu, Yanhui Sheng, Wei Sun, Xiangqing Kong

**Affiliations:** ^1^ Department of Cardiology The First Affiliated Hospital of Nanjing Medical University Nanjing China; ^2^ Cardiovascular Device and Technique Engineering Laboratory of Jiangsu Province Nanjing China; ^3^ State Key Laboratory of Reproductive Medicine Nanjing Medical University Nanjing China

**Keywords:** cardiac fibrosis, histone deacetylase 1, peptidase inhibitor 16

## Abstract

Cardiac hypertrophy and fibrosis are the major causes of heart failure due to non‐ischaemia heart disease. To date, no specific therapy exists for cardiac fibrosis due to the largely unknown mechanisms of disease and lack of applicable therapeutic targets. In this study, we aimed to explore the role and associated mechanism of peptidase inhibitor 16 (PI16) in cardiac fibrosis induced by angiotensin II. In cardiac fibroblasts (CFs), overexpressed PI16 significantly inhibited CF proliferation and the levels of fibrosis‐associated proteins. Further analysis of epigenetic changes in CF revealed that overexpressed PI16 decreases the nuclear level of histone deacetylase 1 (HDAC1) after angiotensin II treatment, resulting in increased histone 3 acetylation in K18 and K27 lysine. However, overexpression of HDAC1 by an adenovirus vector in CFs reversed these changes. Echocardiography showed that PI16 transgenic (Tg) mice have smaller left ventricle mass than wild‐type mice. Histological analysis data showed that PI16 Tg mice demonstrated smaller cardiomyocyte size and less collagen deposition than wild‐type mice. The effects of PI16 on HDAC1 and histone 3 were also confirmed in PI16 Tg mice using immunostaining. Generally, PI16 is a HDAC1 regulator specifically in CFs, and PI16 overexpression prevents cardiac hypertrophy and fibrosis by inhibiting stress‐induced CF activation.

## 1 INTRODUCTION

Hypertension and chronic heart failure have become major health problems worldwide, especially in developing countries.^1^ Cardiac hypertrophy and fibrosis are critical pathological processes associated with hypertension and heart failure.^2^ Cardiomyocytes (CMs) are the predominant cellular components responsible for cardiac contractility. However, cardiac fibroblasts (CFs) constitute 72% of total heart cells in humans, 64% in rats and 27% in mice.^3^, ^4^ Under normal physiological conditions, CFs are the major producers of the extracellular matrix and contribute to a mechanical scaffold for cardiomyocyte contractions. During the process of increased stress including pressure overload and myocardial infarction, CF provides a pivotal contribution to cardiac remodelling via the secretion of many growth factors and extracellular matrix components, as well as cell proliferation. However, no specific therapy for cardiac fibrosis currently exists largely because the underlying basis of cardiac fibrosis is complicated and not fully understood.

With the discovery of multiple mechanisms mediating the development of cardiac fibrosis, it has become clear that many of the signalling pathways, such as those mediated by TGF‐β and angiotensin II (Ang II), are highly redundant. Because functional redundancy cannot be a realistic therapeutic target, researchers were prompted to search for the common downstream mediators to serve as pharmacological targets. Recent studies suggest that histone deacetylases (HDACs), particularly class I HDACs, which primarily deacetylate histones involved in various cellular biological processes,^5^, ^6^ may represent such targets. In animal studies, small‐molecule HDAC inhibitors such as trichostatin A, MGCD0103 and apicidin derivatives potently attenuate cardiac fibrosis by suppressing cardiac fibroblast proliferation in response to diverse upstream signalling cascades.^7^, ^8^, ^9^

Peptidase inhibitor (PI16), first identified from prostate tumour tissues, functions as a binding partner for a prostate secretory protein of 94 amino acids (PSP94) in patients with prostate cancer and is reported to have potentially prognostic values.^10^, ^11^ Frost et al first reported that PI16 is highly expressed in the heart and involved in isoproterenol‐induced cardiac hypertrophy.^12^ A gene expression profiling study also revealed the involvement of PI16 in cardiac fibrosis.^13^ Another study found that PI16 is highly expressed in regulatory T cells and enhances their chemoattraction.^14^ A recent study reported that whole‐tissue deletion of PI16 showed no effect on cardiac hypertrophy induced by pressure overload and that cardiac fibroblast–secreted PI16 suppresses stress‐induced chemerin activation in the myocardium.^15^ However, the role of PI16 in CFs during pathological cardiac hypertrophy and fibrosis remains unknown.

In this study, cellular experiments demonstrated that PI16 overexpression attenuates CF proliferation, activation and collagen synthesis via down‐regulating HDAC1. We generated pan‐expressional PI16 transgenic (PI16‐Tg) mice using an EF1α‐promoter and found that PI16 is primarily overexpressed by CFs. Using the Ang II–induced cardiac hypertrophy and fibrosis model, we found that PI16 overexpression inhibited cardiac hypertrophy and collagen synthesis induced by Ang II.

## 2 MATERIALS AND METHODS

### 2.1 Animal care and study approval

PI16‐Tg mice on the FVB background were generated by Cyagen Biosciences Inc through microinjection with a recombinant PI16 vector containing an elongation factor 1 α (EF1‐α) promoter. Mice were maintained under specific pathogen‐free conditions and mated with C57BL/6J mice. PCR genotyping used the primers PI16 forward 5′‐CAAGTTTGTACAAAAAAGCAGGCT‐3′ and reverse 5′‐CACTTTGTACAAGAAAGCTGG‐3′ to generate 1550‐bp PI16 fragments. All experiments were approved by the Animal Ethical and Welfare Committee of Nanjing Medical University. All mice were raised at the animal core facility of Nanjing Medical University. Mice were observed daily, and the cages were changed twice a week. All mice had free access to water and standard chow.

### 2.2 Ang II–induced mouse model

Eight‐ to twelve‐week‐old male PI16‐Tg mice and their wild‐type (WT) littermates (control) were subjected to Ang II (3.6 mg/kg/d, Sigma‐Aldrich) or stroke‐physiological saline solution (control) treatment; each of the four groups contained 9‐11 mice. Ang II was diluted with saline solution and continuously pumped for 28 days via mini‐osmotic pumps (Alzet, 2004). The pumps were subcutaneously implanted after anaesthetizing mice with 3.5% chloral hydrate. For analysis of echocardiographic assessment, a Vevo 2100 instrument (VisualSonics) was used after 4 weeks of Ang II treatment. At the end of 4 weeks of treatment, animals weighed and then killed under anaesthesia with 3.5% chloral hydrate. Hearts were harvested, washed in PBS, dried and weighed, and photographed by a microscope (Carl Zeiss). Part of the tissue was frozen to −80°C for further Western blot or PCR analysis. Another part was fixed with fresh diluted 4% paraformaldehyde overnight at 4°C before being embedded and frozen in optimal cutting temperature compound (Tissue‐Tek) for immunohistochemical or immunofluorescence staining. In addition, the tibia was removed, and the length was measured by a Vernier calliper. Ratios of heart weight to tibial length (HW/TL) and heart weight to bodyweight (HW/BW) were calculated then.

### 2.3 Immunohistochemical staining

Sirius red, haematoxylin‐eosin and Masson's trichrome staining were used, following the appropriate methods for histological techniques. Haematoxylin‐eosin and trichrome images of the Ang II models were captured with an Axio Scan Z.1 microscope (Carl Zeiss). Other images were captured by a fluorescence microscope (Carl Zeiss). Images were analysed with ImageJ software.

### 2.4 Western blot

For protein extraction, 10 mg heart tissue was homogenized in 100 µL RIPA lysis buffer (Thermo Scientific) supplemented with protease inhibitor (Thermo Scientific) and phosphatase inhibitor (Roche) cocktail. Cell proteins were extracted with lysis buffer (KeyGEN BioTECH) supplemented with a protease/phosphatase inhibitor cocktail. Nuclear proteins were obtained with the Nucbuster Protein Extraction Kit (Millipore #71183‐3) used according to the manufacturer's protocol. Protein concentration was assayed with the Pierce BCA protein assay kit (Thermo Scientific). Prior to Western blotting, 10%‐15% SDS‐PAGE was prepared. After electrophoresis and transfer to polyvinylidene difluoride membrane (Millipore), the membranes were blocked with 5% bovine serum albumin solution. Then, the membranes were incubated with primary antibodies overnight at 4°C and HRP‐linked secondary antibodies (1:5000) for 2 hours at room temperature. The following primary antibodies were used: anti‐collagen‐I (1:500; Wanleibio #WL0088), anti‐phospho‐Akt (Ser473) (1:1000; Cell Signaling #4060), anti‐Akt (pan) (1:1000; Cell Signaling #4691), anti‐PI16 (1:500, Atlas Antibodies #HPA043763), anti‐p53 (1:1000; Cell Signaling #2524), anti‐acetyl‐p53 (Lys382) (1:1000; Cell Signaling #2525), anti‐alpha smooth muscle actin (1:200; Abcam #ab5694), anti‐TGF‐β (1:1000; Cell Signaling #3711), anti‐phospho‐Smad2 (Ser465/467)/Smad3 (Ser423/425) (1:1000; Cell Signaling #9510), anti‐GAPDH (1:1000; Cell Signaling #5174), anti‐histone H3 (1:1000; Cell Signaling #9717), anti‐acetyl‐histone H3 (1:1000; Millipore #17‐615), anti‐acetyl‐histone H4 (1:1000; Millipore #06‐598), anti‐P300 (1:200; Santa Cruz sc‐585), anti‐CBP (1:200; Santa Cruz sc‐369) and anti‐GCN5 (1:200; Santa Cruz sc‐20698). Protein levels of HDACs were assessed by Histone Deacetylase Antibody Sampler Kit (1:1000; Cell Signaling #9928). Levels of acetyl‐histone H3 were assessed by Acetyl‐Histone H3 Antibody Sampler kit (1:1000; Cell Signaling #9927). The blots were scanned with a ChemiDoc MP imager (Bio‐Rad). Images were quantified by Image Lab™ software.

### 2.5 Cell isolation and culture

Newborn Sprague‐Dawley rats (1‐3 days old) and Adult Sprague‐Dawley rats (6 weeks old) were obtained from the Center of Experimental Animals of Nanjing Medical University. Newborn rat ventricular myocytes (NRVMs) and cardiac fibroblasts (NRCFs) and adult rat cardiac fibroblasts (ARCFs) were isolated and cultured using previously described methods with some modifications.^16^, ^17^ Briefly, the hearts from newborn rats were quickly removed before the rats were killed. Ventricles were washed with ADS (NaCl, HEPES, NaHPO_4_, glucose, KCl and MgSO_4_·7H_2_O diluted in ddH_2_O; all reagents from Sigma‐Aldrich) and cut into small chunks. Then, the tissue was digested with type 2 collagenase (Worthington) and pancreatin (Sigma‐Aldrich) at 37°C for 10 minutes with rotation. The digestion was stopped by horse serum (Gibco), and cells were then centrifuged at 160.99 x *g* for 3 minutes and re‐suspended in Dulbecco's modified Eagle's medium (DMEM) supplemented with penicillin, streptomycin and 10% foetal bovine serum (Gibco). This digestion procedure was repeated until most of the cells had been released from ventricular tissue. Cells were then seeded into 10‐cm culture dishes (Corning) and incubated at 37°C in a 5% CO_2_ incubator. After 1 hour, attached cells (NRCFs or ARCFs) were cultured further with DMEM supplemented with 10% FBS. Passages 3‐5 of normal NRCF or ARCF cells were used for further experiments. Unattached cells were collected and separated using a Percoll gradient (Sigma‐Aldrich). NRCMs were seeded into 6‐cm culture dishes (Corning) after cell concentration was adjusted. NRVMs were cultured in DMEM supplemented with 10% horse serum, 5% FBS and 1% penicillin‐streptomycin at 37°C in a 5% CO_2_ incubator.

### 2.6 Real‐time PCR

An RNeasy RNA Isolation Kit (Qiagen) was used to extract RNA of NRCFs and mouse hearts. A PrimeScript™ RT Reagent Kit (Takara) was used to perform reverse transcription. After normalizing the primer concentrations and mix gene‐specific forward and reverse primer pairs (5 pmol/µL), cDNA was used for PCR (ABI Prism 7900 system). Genes were detected by TaqMan probes (Roche). Each sample was analysed in triplicate, and target genes were normalized to the housekeeping gene glyceraldehyde 3‐phosphate dehydrogenase (GAPDH). Fold differences were then calculated for each treatment group after ΔΔCt correction. The following primer sets were used to identify transcripts: myocyte enhancer factor 2 (MEF2c), 5′‐ATCCCGATGCAGACGATTCAG and 5′‐AACAGCACACAATCTTTGCCT; nuclear factor of activated T cells 1 (NFATc1), 5′‐GGAGAGTCCGAGAATCGAGAT and 5′‐TTGCAGCTAGGAAGTACGTCT; nuclear factor of activated T cells 2 (NFATc2), 5′‐CGGGCTCCTATGAGCTACG and 5′‐CTCGTGGTGGTGACCGTTTT; atrial natriuretic peptide (ANP), 5′‐GTGCGGTGTCCAACACAGAT and 5′‐TCCAATCCTGTCAATCCTACCC; brain natriuretic peptide (BNP), 5′‐GAGGTCACTCCTATCCTCTGG and 5′‐GCCATTTCCTCCGACTTTTCTC; collagen Ia1, 5′‐GCTCCTCTTAGGGGCCACT and 5′‐CCACGTCTCACCATTGGGG; collagen III, 5′‐CGAGATTAAAGCAAGAGGAA and 5′‐GAGGCTTCTTTACATACCAC; PI16, 5′‐TCAGACATGCTGCAGATGAGGTGGG and 5′‐AGCCAAGAGGGCACTGGGAGCAAGG; HDAC1, 5′‐TGAAGCCTCACCGAATCCG and 5′‐GGGCGAATAGAACGCAGGA; p21 (promotor), 5′‐TGTAAGGGCTGTGACATTGC and 5′‐GCCTGAGAGGATTTCAATGG; GAPDH, mouse, 5′‐GCTCCTCTTAGGGGCCACT and 5′‐TGTAGACCATGTAGTTGAGGTCA; and GAPDH, rat, 5′‐GGCACAGTCAAGGCTGAGAATG and 5′‐ATGGTGGTGAAGACGCCAGTA.

### 2.7 Angiotensin II treatment in vitro

To induce fibrosis and hypertrophy in vitro, NRCFs or NRVMs were treated with 1 μmol/L Ang II (Sigma‐Aldrich) diluted in DMEM (Gibco) for 24 hours. To examine the paracrine effect of PI16 from NRCFs in vitro, NRVMs were treated with 1 μmol/L Ang II (Sigma‐Aldrich) which was diluted in conditioned medium of NRCFs which were infected with Ad‐GFP or Ad‐PI16 for 24 hours.

### 2.8 Immunofluorescence

Frozen tissue was sectioned by a cryostats (Leica) at a thickness of 7 μm for immunofluorescent staining. Samples were fixed in fresh diluted 4% paraformaldehyde for 10 minutes and washed in PBS. Tissue sections were permeabilized with 0.05% Triton X‐100 for 10 minutes and washed in PBS. After blocking with a solution (5% bovine serum albumin and 10% goat serum in PBS) for 1 hour at room temperature, tissue sections were incubated with primary antibodies overnight at 4°C and then secondary antibodies for 1 hour at room temperature. The following primary antibodies were used: anti‐PI16 (1:50; Cloud‐Clone Corp #PAQ943Mu01), anti‐HDAC1 (1:100; GeneTex #GTX100513), anti‐acetyl‐histone H3 (1:100; Millipore #17‐615), anti‐acetyl‐histone H3 (Lys18) (1:100; Cell Signaling #13998), anti‐acetyl‐histone H3 (Lys27) (1:100; Cell Signaling #8173) and anti‐vimentin (1:100; Abcam #ab92547). The secondary antibodies used were conjugated to Alexa‐488 (1:400; Jackson #111‐545‐003) or CyTM3 (1:400; Jackson #111‐165‐003). The nuclei were stained with 4′,6‐diamidino‐2‐phenylindole (DAPI) for 10 minutes at room temperature. Tissue sections were covered with cover slips and anti‐fade mounting media (Invitrogen). Confocal laser scanning microscopy (Carl Zeiss) was used to scan samples.

### 2.9 Adenovirus transfection

Cells were cultured to a density of 60%‐70% before adenovirus transfection. According to the manufacturer's instructions, NRCFs or NRVMs were infected with recombinant adenovirus expressing PI16 (0.8 × 10^7^ pfu/mL) or short hairpin RNAs targeting PI16 (10 × 10^7^ pfu/mL), HDAC1 (0.5 × 10^7^ pfu/mL) or GFP (1.0 × 10^7^ pfu/mL) for controls. Adenovirus was purchased from GeneChem.

### 2.10 Real‐Time Cellular Analysis (RTCA)

Proliferation of NRCFs transfected with Ad‐GFP, Ad‐PI16 or Ad‐PI16 + Ad‐HDAC1 was determined with the xCELLigence™ RTCA Instrument from ACEA. Approximately 2000 NRCFs in complete medium were seeded into each well (5‐6 wells/group) of xCELLigence E‐Plate 16(Roche) placed on the sensor. Cell index was recorded every 15 minutes.

### 2.11 5‐Ethynyl‐2′‐deoxyuridine (Edu) staining

After adenovirus transfection for 3 days, NRCFs were cultured with 20 µmol/L Edu (Invitrogen) for 24 hours. Wash the cells for 3 times with 1× PBS. NRCFs were fixed with fresh diluted 4% paraformaldehyde for 20 minutes, washed with 3% BSA, permeabilized with 0.5% Triton X‐100 at RT for 10 minutes. After being washed with 3% BSA for 3 times, NRCFs were incubated with Alexa Flour^®^ 488 solution (1:400, Invitrogen) at RT for 1 hour in the darkness and then with Hoechst 33342 (1:2000, Invitrogen) for 5 minutes. NRCFs were visualized and photographed with a fluorescence microscope (Carl Zeiss).

### 2.12 Evaluation of cardiomyocyte hypertrophy

Newborn rat ventricular myocytes were seeded onto an 8‐well chamber slide (Thermo Scientific) at a confluence of 40%‐50% before treatments. After Ang II treatment for 24 hours, NRVMs were fixed with fresh diluted 4% paraformaldehyde for 10 minutes and permeabilized with 0.05% Triton X‐100 for 10 minutes. After blocking with 5% bovine serum albumin and 10% goat serum in PBS for 1 hour at room temperature, NRVMs were incubated with anti‐sarcomeric alpha actinin antibodies (1:600; Abcam #ab137346) overnight at 4°C and then goat anti‐mouse secondary antibody conjugated to CyTM3(1:400; Jackson #111‐165‐003) for 1 hour at room temperature. The nuclei were labelled with DAPI and covered with cover slips and anti‐fade mounting media (Invitrogen), and then, NRVMs were visualized and photographed with a fluorescence microscope (Carl Zeiss). Cell size was analysed with ImageJ software.

### 2.13 Chromatin immunoprecipitation (ChIP) assay

NRCFs were fixed with 1% paraformaldehyde at RT for 10 minutes and 125 mmol/L glycine solution at RT for 3 minutes with rotation. After washed with cold 1× PBS, NRCFs were collected with protease inhibitor buffer (1× protease inhibitor + 10 mL 1× PBS) and then centrifuged at 600.66 x *g* for 10 minutes at 4°C. The cells were then frozen overnight at −80℃. The cells were lysed with lysis buffer (1× protease inhibitor + 10 mL cell lysis buffer) and then centrifuged at 2500 rpm for 5 minutes at 4°C. The sediment was re‐suspended with nucleus lysis buffer (1× protease inhibitor + 10 mL nucleus lysis buffer). After sanitation, the lysates were centrifuged at 13 839.11 x *g* for 10 minutes at 4°C. 10% of the samples were used as input and were frozen at −80℃. Then, the residual samples were pre‐cleaned in dilution buffer (dilution buffer 10 mL + 1× protease inhibitor) and incubated with magnetic beads and indicated antibodies or IgG overnight at 4℃. The magnetic beads were washed with low salt, high salt, LiCl wash buffer and TE buffer (all from MagnaChIP A/G 17‐10085 sample kit). The binding components were then incubated with elusion buffer and 10 µL 5 mol/L NaCl and performed reverse cross‐link at 65℃ overnight. The samples were incubated with 1 µL RNaseA at 37℃ for 1 hour and then with 0.5 mol/L EDTA + 2 µL 10 mg/mL PK + 10 µL 1 mol/L Tris‐HCl (pH 6.5) at 45℃ for 2 hours. DNA was extracted by PCR Purification Kit (Qiagen, 28006). Real‐time PCR was used to detect relative enrichment of the modification on indicated genes.

### 2.14 Statistical analysis

All measurement data are shown as the means ± standard errors of the mean (SEM). Treatment group values were compared with control values using GraphPad Prism 6.0 (GraphPad Software). Depending on the type of data, one‐way ANOVA followed by Bonferroni's multiple comparison test or Student's *t* test was used to determine statistical differences. *P *values <.05 were considered to be statistically significant.

## 3 RESULTS

### 3.1 Overexpression of PI16 inhibits cardiac fibroblast proliferation

**Figure 1:**
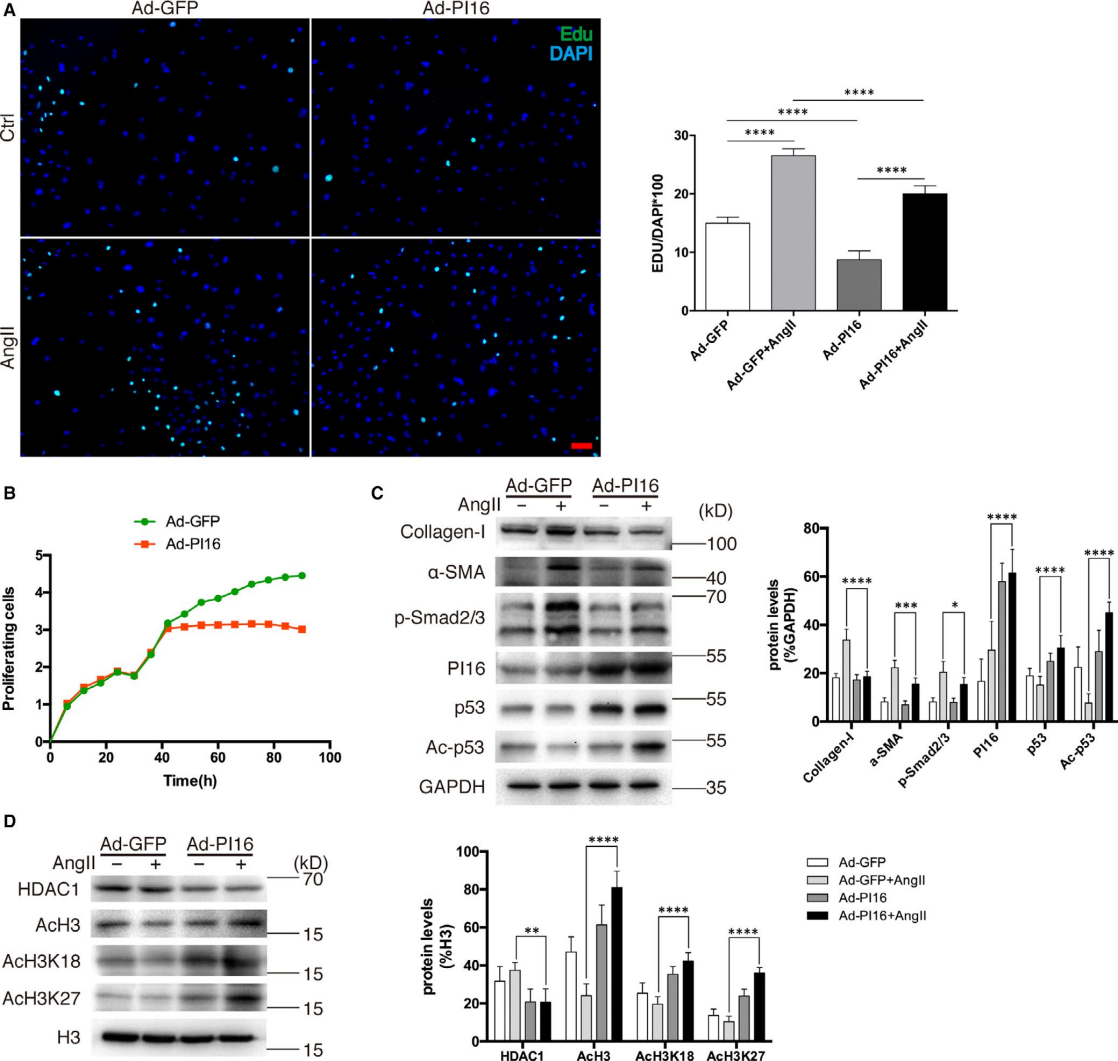
Overexpression of peptidase inhibitor 16 (PI16) attenuates angiotensin II (Ang II)–induced fibrotic gene expression and histone 3 modification in NRCFs. A, Representative images and quantitation of Edu‐labelled (green) NRCFs with Ad‐PI16 or Ad‐GFP transfection and Ang II treatment. Scale bar, 50 μm. B, Cell proliferation was assessed by a real‐time cell proliferation assay (RTCA) using electric impedance as a measure of proliferation in NRCFs transfected with Ad‐PI16 or Ad‐GFP every 15 min. Electrical impedance was normalized according to the background measurement at time point 0. C, Protein levels of collagen I, α‐smooth muscle actin, p‐Smad2/3, PI16, p53 and Ac‐p53 in newborn rat cardiac fibroblasts (NRCFs) were assessed by Western blotting. The relative protein levels were normalized to GAPDH. D, Nuclear protein levels of HDAC1, AcH3, AcH3K18 and AcH3K27 were assessed by Western blotting. The relative protein levels were normalized to histone 3 (H3). Data are shown as the means ± standard errors of the mean of triplicates and are representative of three independent experiments performed. *P < .05, **P < .01, ***P < .001, ****P < .0001

To examine the role of PI16 in cardiac fibroblast in vitro, we isolated cardiac fibroblasts from Newborn Sprague‐Dawley rats (NRCFs) and transfected NRCFs with adenovirus PI16 vector (Ad‐PI16) or adenovirus GFP vector (Ad‐GFP) for further experiments. Edu staining and RTCA assays demonstrated that overexpression of PI16 significantly inhibited cell proliferation of NRCFs (Figure 1A‐C). After starvation for 6‐8 hours, NRCFs were treated with 1 µmol/L angiotensin II (Ang II). We found that overexpressed PI16 in NRCFs reduced Ang II–induced cell proliferation of NRCFs and pro‐fibrotic protein expression including collagen I, α‐smooth muscle actin and p‐Smad2/3 (Figure 1A,C). We further examined the cell cycle gene and found that overexpressed PI16 increased p53 expression in NRCFs (Figure 1C), suggesting that p53 may be a mediator for the anti‐proliferation effect of PI16. To further determine the role of PI16 in NRCF cellular function, we knock down PI16 by transfecting NRCFs with recombinant adenovirus expressing short hairpin RNAs targeting PI16 (Figure S1A). RTCA assay showed no difference in cell proliferation between PI16 knocking down and control groups (Figure S1B). Edu and Ki67 staining indicated that Ang II treatment promoted proliferation of NRCFs, and PI16 down‐regulation did not show any influence on these changes (Figure S1C,D). Moreover, PI16 knock‐down did not affect the expression of pro‐fibrotic markers by Ang II (Figure S1E). To examine the role of PI16 in adult fibrosis, we isolated cardiac fibroblasts from adult rats (ARCFs) and overexpressed PI16 with Ad‐PI16 or knock‐down PI16 with Ad‐RNAiPI16 for the further experiments. After treated with 1 µmol/L Ang II, we assessed pro‐fibrotic and cell cycle–related protein expression by Western blotting. Similar to the results of NRCFs, overexpression of PI16, not knock‐down of PI16, attenuated Ang II–induced fibrotic gene expression in ARCFs (Figure S2). Overexpressed PI16 increased p53 expression in ARCFs, indicating that PI16 also plays an anti‐proliferation role in ARCFs (Figure S2A).

### 3.2 HDAC1 inhibition by PI16 blocks fibrosis in vitro

Recent studies have shown that altered lysine acetylation, by targeting HDACs, has the remarkable ability to block pathological fibrosis, and class I HDACs play roles in pathological cardiac fibrosis.^18^, ^19^ To find out why PI16 inhibits Ang II–induced pro‐fibrotic changes in NRCFs, we examined the protein levels of histone acetyltransferase (HAT) and HDACs in NRCFs after Ang II treatment. We found that PI16 overexpression significantly decreased HDAC1 levels and selectively increased acetylation of histone H3 (AcH3) (Figure 1E and Figure S3A,C). Previous studies have indicated that HDAC1 is a negative regulator of p53 and associated with p53 deacetylation and degradation,^20^, ^21^, ^22^, ^23^ and we examined the levels of acetylated p53. Our results showed that the acetylation of p53 was increased in PI16 overexpressed NRCFs after Ang II treatment (Figure 1C). After examining the acetylation levels of different lysine sites of H3, we found that PI16 overexpression increased H3K18 and H3K27 acetylation in NRCFs (Figure 1D) but did not affect H3K9, H3K14, H3K56 or pan‐H4 acetylation (Figure S3B,D). These data suggest that PI16 may inhibit Ang II–induced fibrotic phenotype in NRCFs via increasing acetylation of H3K18 and H3K27, and p53 stability by decreasing HDAC1.

### 3.3 Overexpression of HDAC1 rescues the effect of PI16 in NRCFs

**Figure 2:**
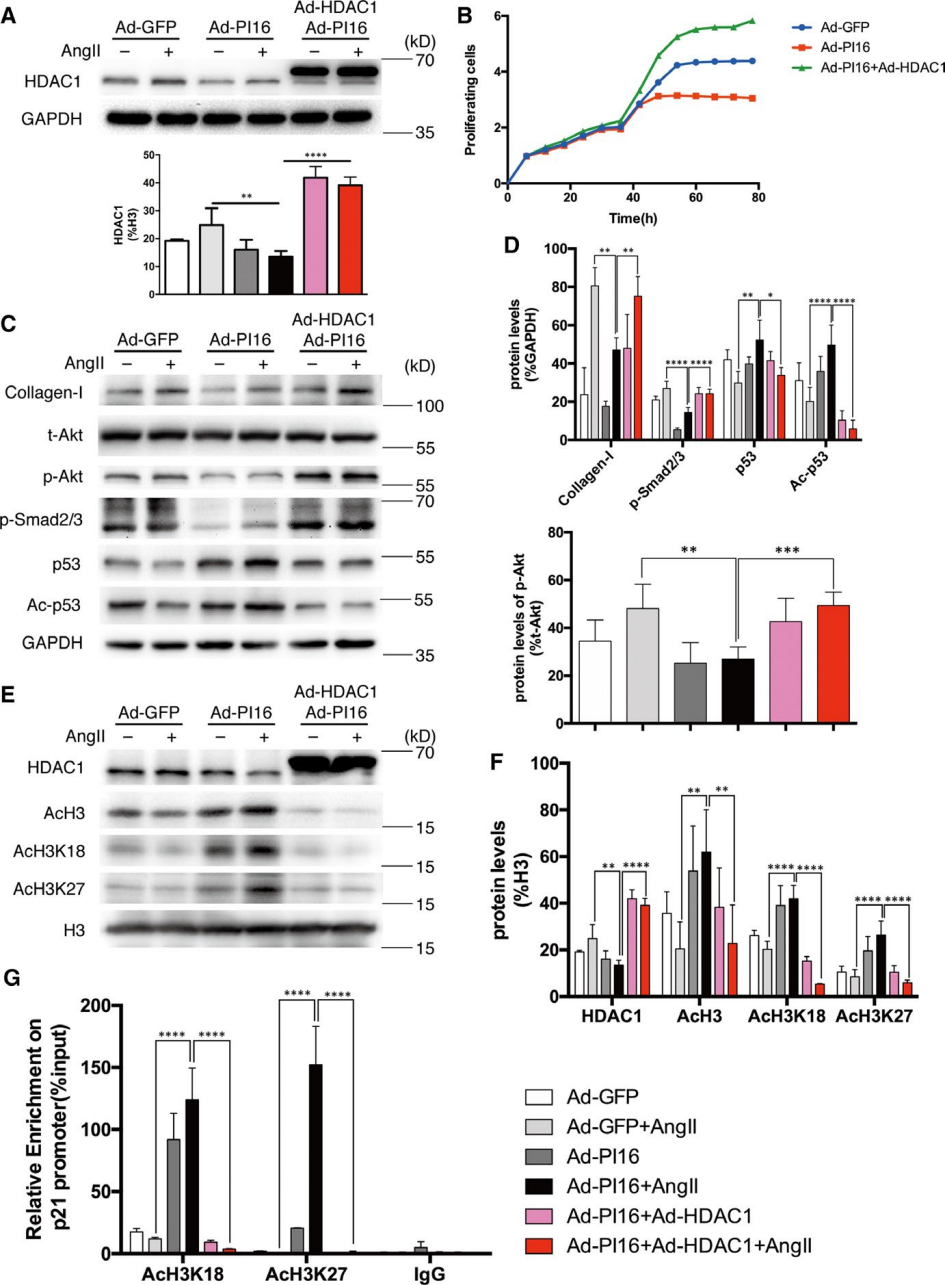
The effects of peptidase inhibitor 16 (PI16) on newborn rat cardiac fibroblasts (NRCFs) are partially rescued by overexpression of HDAC1. A, Overexpression of HDAC1 was assessed by Western blotting. The relative protein levels were normalized to GAPDH. B, NRCF proliferation was assessed by RTCA every 15 min. Electrical impedance was normalized according to the background measurement at time point 0. C and D, The expression of collagen I, t‐Akt, p‐Akt, p‐Smad2/3, p53 and Ac‐p53 was assessed by Western blotting. The relative protein levels were normalized to GAPDH; protein levels of p‐Akt were normalized to t‐Akt. E and F, Nuclear protein levels of HDAC1, AcH3, AcH3K18 and AcH3K27 were assessed by Western blotting. Relative protein levels were normalized to histone 3 (H3). G, ChIP analysis of the recruitments of acetylated H3K18 and H3K27 into p21 promoter in NRCFs. Data are shown as the means ± standard errors of the mean of triplicates and are representative of three independent experiments performed. *P < .05, **P < .01, ***P < .001, ****P < .0001

To confirm the hypothesis that decreased HDAC1 contributes to PI16’s effect, we up‐regulated the level of HDAC1 in NRCFs by co‐transfection with Ad‐HDAC1 and Ad‐PI16. Western blotting showed that the molecular weight of exogenous HDAC1 was slightly larger than that of native HDAC1 (Figure 2A). Cell proliferation assays demonstrated that increased HDAC1 reversed this inhibitory effect on cell proliferation by PI16 (Figure 2B). As expected, Western blotting showed that increased HDAC1 reversed PI16‐mediated reduction in collagen I, p‐Smad2/3 and p‐Akt (Figure 2C,D). The total protein and acetylation levels of p53 were also suppressed in response to HDAC1 overexpression (Figure 2C,D). To confirm the role of HDAC1 in pro‐fibrotic procedure, we up‐regulated the level of HDAC1 in NRCFs. Western blotting showed that HDAC1 increased fibrotic markers such as collagen I, TGF‐β and p‐Smad2/3 after Ang II Treatment, but decreased p53 level (Figure S4). Nuclear protein levels showed that increased HDAC1 led to decreased AcH3, AcH3K18 and AcH3K27 (Figure 2E,F). ChIP analysis revealed that PI16 overexpression significantly increased the recruitment of AcH3K18 and AcH3K27 at p21 promoter, while increased HDAC1 reversed these effects (Figure 2G). Hence, these data reveal that HDAC1 inhibition by PI16 prevents fibrotic gene expression via increasing histone 3 acetylation–mediated epigenetic regulation on p21 promoters.

### 3.4 PI16 prevents Ang II–induced cardiac hypertrophy and fibrosis in vivo

**Figure 3:**
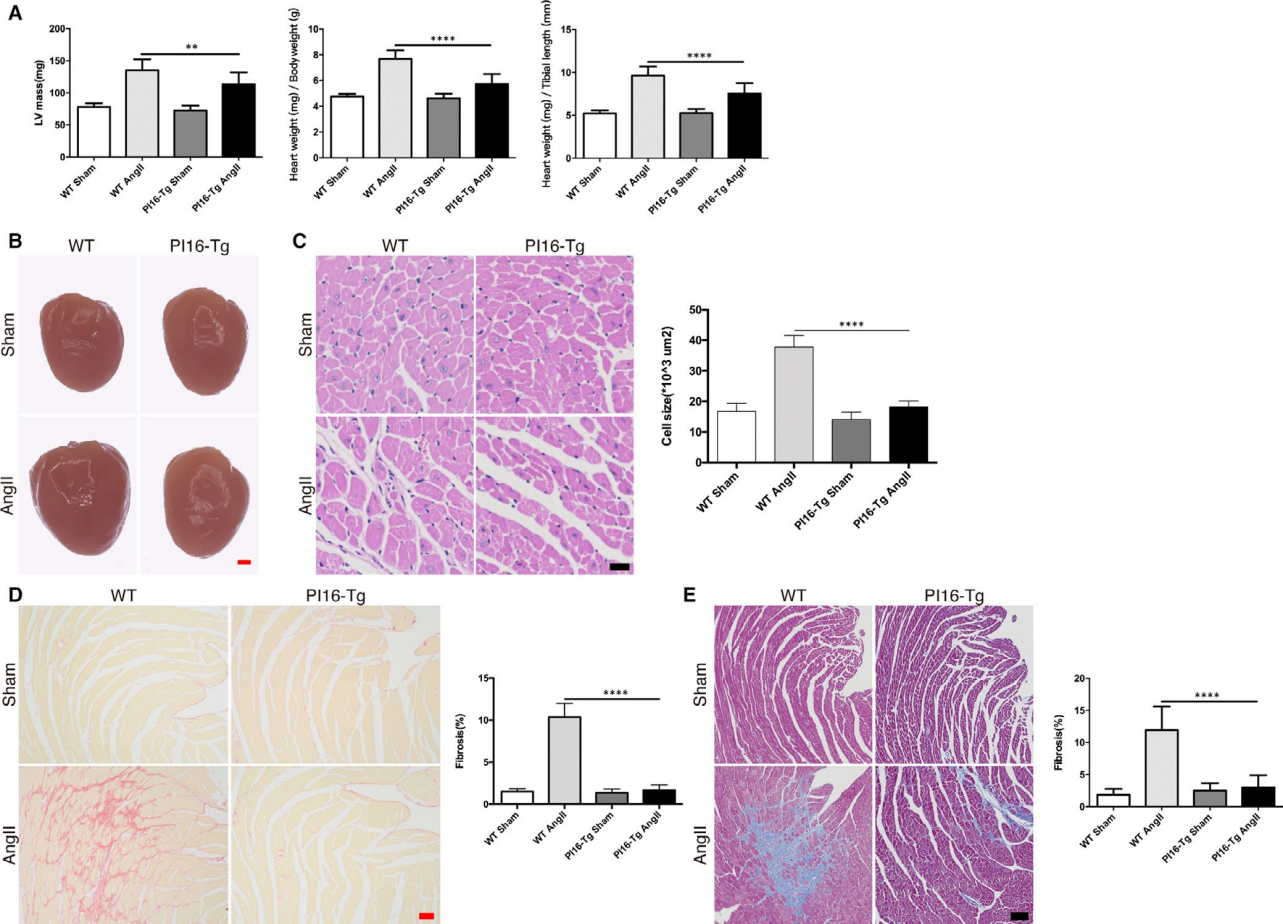
Overexpression of peptidase inhibitor 16 (PI16) reduces cardiac hypertrophy and fibrosis induced by Ang II. A, The ratios of heart weight to tibial length (HW/TL) and heart weight to bodyweight (HW/BW) were calculated, and left ventricle (LV) mass was measured by echocardiography in mice after 4‐wk Ang II treatment. B, Photographs of whole hearts from PI16‐Tg and WT mice treated with or without Ang II. Scale bar, 1 mm. C, Cell size of cardiomyocytes of PI16‐Tg and WT mice treated with or without Ang II was visualized by haematoxylin‐eosin staining and quantified. Scale bar, 20 μm. D, Collagen deposition was measured by Sirius red staining and quantified. Scale bar, 50 μm. E, Collagen deposition was measured by Masson's trichrome–stained and quantified. Scale bar, 100 μm. Data are shown as the means ± standard errors of the mean (SEM) of triplicates and are representative of three independent experiments. n = 9‐11 for each group. **P < .01, ****P < .0001

**Figure 4:**
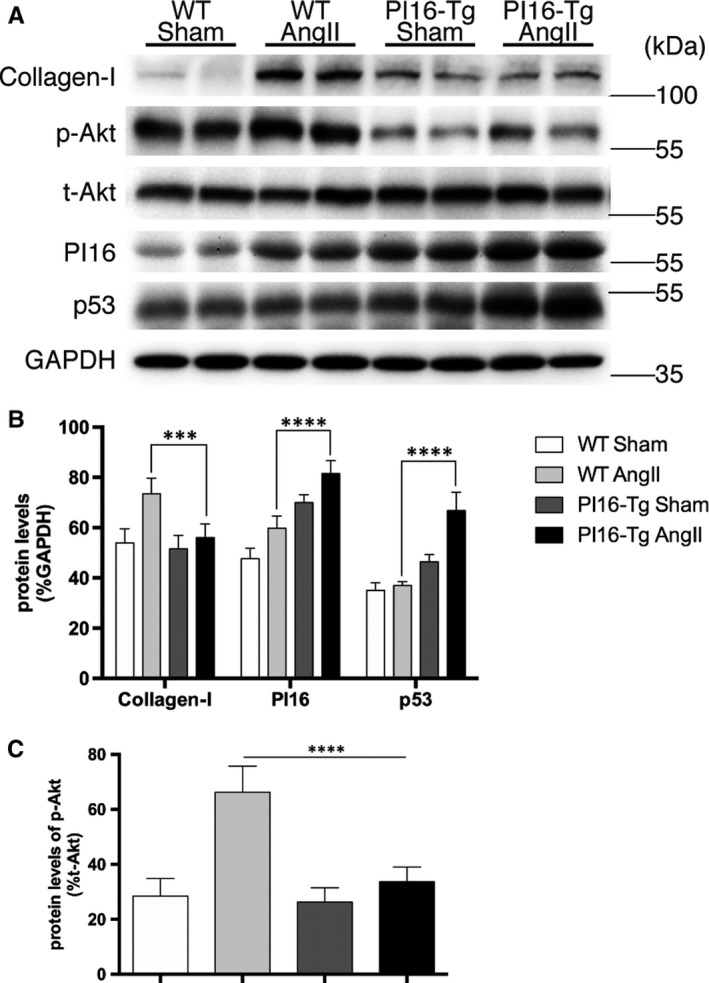
A, Protein levels of collagen I, p‐Akt, t‐Akt, PI16, and p53 in ventricular tissues from wild‐type (WT) and PI16 transgenic (PI16‐Tg) mice treated with or without Ang II were assessed by western blotting. B, Quantification of relative protein levels were normalized to GAPDH. C, Protein levels of p‐Akt were normalized to t‐Akt. Data are shown as the means ± standard errors of the mean of triplicates and are representative of three independent experiments performed. N = 9‐11 for each group. ****P < .0001

To further confirm the role of PI16 in cardiac remodelling in vivo, we study the role of PI16 in a PI16 transgenic mouse model (PI16‐Tg) that PI16 was successfully overexpressed in heart tissues (Figure S5). Immunostaining showed that PI16 is mainly expressed by CFs both in PI16‐Tg and WT mice (Figure S5). We then build up a classical cardiac hypertrophy and fibrosis model in PI16‐Tg mice and their WT littermates (control) with continuous 4‐week infusion of Ang II as previously described.^24^ Echocardiography at 4 weeks after Ang II treatment showed that PI16 overexpression decreased the degree of cardiac hypertrophy, but not left ventricle systolic function (Figure 3A and Table S1). The size of heart and cardiomyocytes (CMs) and hypertrophic marker gene including NFAC1 and MEF2C from Ang II–treated PI16‐Tg mice were smaller than those of Ang II–treated WT mice (Figure 3B,C and Figure S6A). However, immunostaining and quantitative PCR showed that PI16 overexpression in NRCMs had no obvious effect on cardiomyocyte hypertrophy (Figure S6B,C), indicating that the anti‐hypertrophy effects of PI16 do not originate from CMs. Recent study has demonstrated that the dominant effects of PI16 on cardiac function of may originate from CFs.^15^ To examine whether overexpression of PI16 in CFs performs paracrine effects, we treated NRVMs with Ang II and conditioned medium from NRCFs transfected with Ad‐PI16 or Ad‐GFP. Immunostaining and quantitative PCR showed that secreted component of PI16 overexpressed CFs significantly attenuated angiotensin II–induced cardiomyocyte hypertrophy (Figure S6D,E), suggesting that the anti‐hypertrophy effects of PI16 may come from CFs. As assessed by Sirius red staining and Masson's trichrome staining, PI16 overexpression decreased Ang II–induced collagen deposition (Figure 3D,E). The protein levels of fibrosis‐related genes, such as collagen I, were lower in Ang II–treated PI16 mice than in Ang II–treated WT mice (Figure 4). PI16 also decreased the activation of Akt, but increased the p53 protein level after Ang II treatment (Figure 4). These results indicate that overexpression of PI16 attenuates Ang II–induced cardiac hypertrophy and fibrosis in vivo.

**Figure 5:**
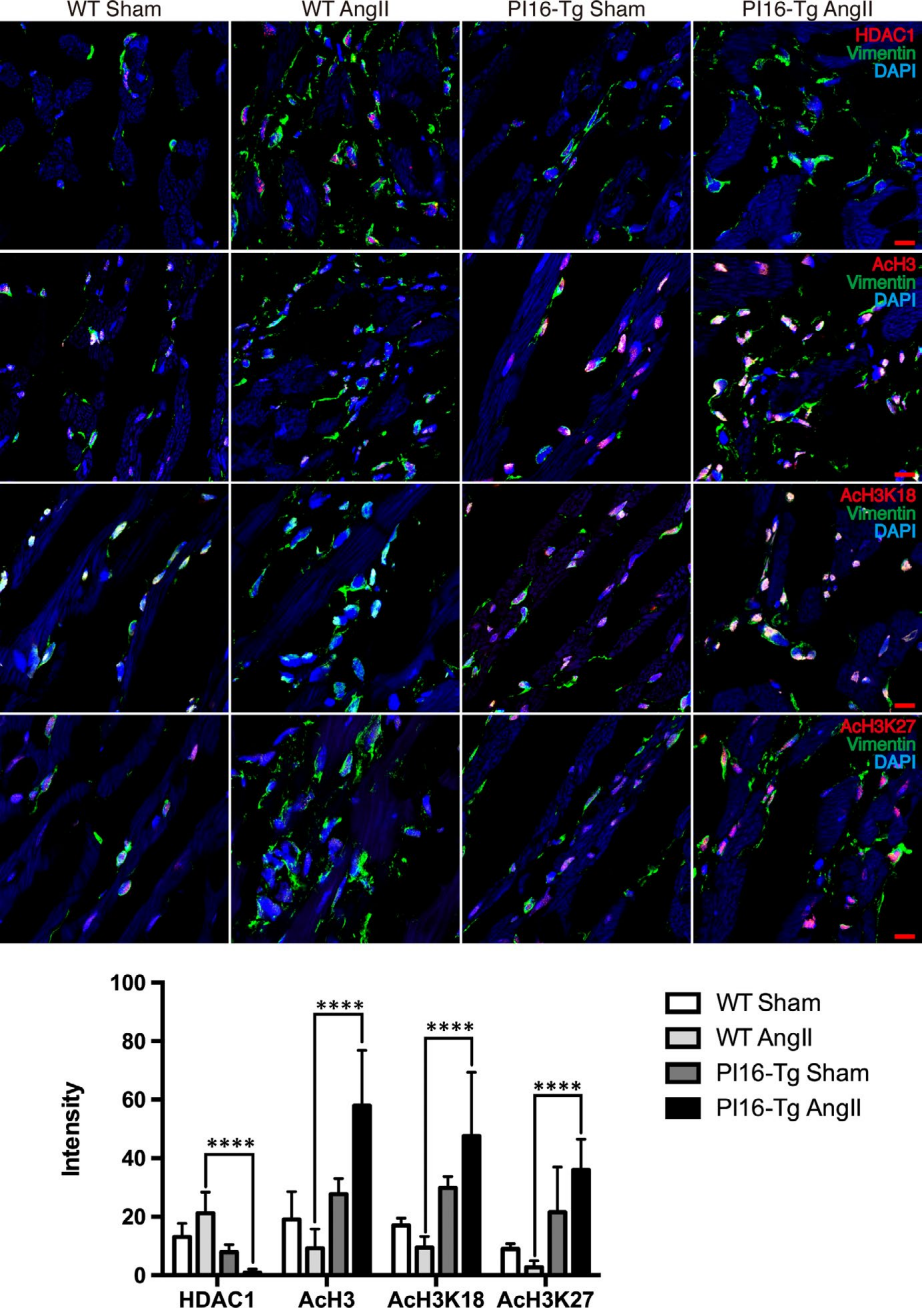
The levels of HDAC1 and histone 3 acetylation in cardiac fibroblasts in vivo. Representative images of immunofluorescent staining and quantitation of HDAC1, AcH3, AcH3K18 and AcH3K27 (red) along with vimentin (green) from ventricular tissues of the indicated genotypes of mice after 4 weeks of angiotensin II treatment. Quantifications of positive cell intensity were normalized to total cells. Scale bar, 20 μm. Data are shown as the means ± standard errors of the mean of triplicates and are representative of three independent experiments performed. ****P < .0001

We further examined the levels of HDAC1, AcH3, AcH3K18 and AcH3K27 in Ang II–treated mice. In agreement with the results in NRCFs, immunofluorescent staining showed that the increased HDAC1 and reduced AcH3, AcH3K18 and AcH3K27 induced by Ang II were blunted in PI16‐Tg mice (Figure 5). These observations support the role of HDAC1 in the anti‐fibrotic effect of PI16 in CFs.

## 4 DISCUSSION

Although significant progress has been made to understand the pathological changes in CM structure and function in different cardiovascular diseases, it has only recently been proved that CFs involved in cardiac fibrosis act as the “elephant in the room” during cardiac hypertrophic or dilated remodelling.^25^ However, the role of CFs in communicating with CMs to regulate cardiac hypertrophy and fibrosis is not fully understood. The major findings of this study are as follows: (a) PI16 decreases CF proliferation and Ang II–induced fibrotic marker protein expression by regulating HDAC1/p53 signalling; (b) highly expressed PI16 in CFs attenuates cardiac hypertrophy and fibrosis induced by Ang II.

In the current study, we used the same strategy to overexpress PI16 in CMs and CFs. However, the biological effects of PI16 to counteract Ang II, as well as the molecular changes induced by Ang II, were mainly found in CFs, not CMs. Our results suggest that PI16 needs to interact with proteins that are specifically expressed by CFs after Ang II treatment. Considering the anti‐proliferating effect of PI16 on CFs and that CMs are non‐proliferating cells, we speculate that some cell cycle–associated proteins are necessary to mediate the effect of PI16 in Ang II–induced CF activation. Further investigations are needed to confirm this hypothesis. In a recent study, PI16 secreted by CFs inhibited cathepsin K, a chemerin‐activating protease, and resulted in suppressed chemerin activation in the myocardium.^15^ By contrast, our study showed that direct overexpression of PI16 with an adenovirus vector in CMs exhibits no anti‐hypertrophic effect on Ang II treatment, which is not consistent with the reduced cardiac hypertrophy found in PI16‐Tg mice. However, secreted components from CFs overexpressed with PI16 can obviously attenuate Ang II–induced cardiomyocyte hypertrophy. These results suggest that PI16 produced by CMs cannot directly attenuate Ang II–induced cardiac hypertrophy, which meet the hypothesis in recent studies that although PI16 exists in cardiac myocytes and is able to modulate their hypertrophic response, the dominant effects of PI16 on cardiac function may originate from the cardiac fibroblasts.^12^, ^15^ Our results also identified the key role of CFs during Ang II–induced cardiac hypertrophy and fibrosis.

Our data showed that overexpression, not down‐regulation of PI16, affects cell proliferation indicating that PI16 does not directly affect cell cycle gene and is not an essential gene for cell viability. The homozygous PI16 deficiency mice displaying no abnormal phenotype under basal conditions or even 28 days after TAC surgery^15^ support that PI16 may be a stress‐associated protein. On the other hand, the constitutive deletion of the PI16 gene may provoke some compensatory gene expression, as often being observed in many other gene deletion cases.^26^ In this study, we mainly studied the role of cell proliferation and response to Ang II, and the full functions of PI16 including cell apoptosis and migration need further investigations.

As an enzyme, HDAC1 plays an important role in various biological processes, such as cell proliferation and differentiation.^27^ HDAC1 is essential for renin‐angiotensin system genes including those that encode angiotensinogen and type 1 angiotensin II receptors in MDCK cells.^28^ The expression of the *HDAC1* gene is stimulated by growth factors in mouse cells^29^ and controlled by its own product in a negative feedback loop.^30^, ^31^ Ang II also increases HDAC1 expression and decreases acetylated histone H3 protein levels.^32^ Even though the role of HDAC inhibitors on cardiac hypertrophy and fibrosis has been confirmed,^7^, ^8^, ^9^ the non‐specificity of HDAC inhibitors may influence the normal function of some other cells and tissues. The endogenous proteins that can reduce HDAC1 levels are not fully understood. Our data firstly identified that PI16 overexpression blunted the increased HDAC1 levels induced by Ang II. In this study, overexpression of HDAC1 partially rescues the inhibitory effect of PI16 on CF proliferation and cardiac fibrosis. Since inflammation has long been recognized as a key stimulus for the development of cardiac fibrosis,^33^, ^34^ and previous study has shown the effect that PI16 suppresses inflammatory‐associated protein chemerin activation in the myocardium.^15^ We think that HDAC1 is another target of PI16 which is independent of inflammation. Moreover, another recent study supports that class I HDAC inhibition blocks cardiac fibrosis independent of the effects on inflammatory responses.^18^ We also demonstrated that PI16 overexpression specifically decreased the protein levels of HDAC1, not HDAC2 and other HDACs, and resulted in increased AcH3, not AcH4. Although it is unclear how PI16 decreases HDAC1 levels induced by Ang II, our study explores an endogenous mechanism to specifically target HDAC1 as a strategy for clinical disease management rather than all HDACs to avoid adverse effects. As small chemical inhibitor specifically targeting HDAC1 cannot be easily developed, RNA interfering strategy, specific antibody or polypeptides derived from PI16 protein may be the choices for the potential therapeutic strategies of managing clinical cardiac fibrosis in the future.

Besides of its histone modification, HDAC1 can deacetylate some other proteins to affect their functions. In the current study, we found that PI16‐mediated reduction in HDAC1 resulted in increased p53 and acetylated p53, and overexpression of HDAC1 reversed these changes in p53. These findings indicate that p53 is an important downstream gene that contributes to the anti‐fibrotic effects of PI16 in CFs. The roles of p53 in cardiac hypertrophy and fibrosis are various. As a tumour suppressor gene, p53 plays different roles during cardiac hypertrophy and fibrosis in a cell‐context dependent manner. In CMs, p53 expression increases as cardiac hypertrophy worsens to heart failure, and p53 deletion attenuates overexpression of myotrophin‐induced hypertrophy.^35^ Similar to its role in CMs, p53 deletion in endothelial cells prevents cardiac fibrosis and heart failure induced by pressure overload.^36^ In contrast to the role of p53 in CMs and endothelial cells, S100A4 as a marker of fibrosis promotes cardiac fibrosis by decreasing p53 expression and p53‐associated gene expression.^37^ In the current study, PI16 overexpression increased p53 and resulted in decreased CF proliferation and collagen expression. Our data confirm the role of p53 in preventing cardiac fibrosis.

Our results showed that due to decreased HDAC1, H3K18 and H3K27 were the most significant lysine sites of acetylation. Generally, H3K18 acetylation (H3K18ac) and H3K27 acetylation (H3K27ac) are associated with active gene expression. One recent study showed that acetylation of H3K18 and H3K27 resulted in a severe proliferation defect in mouse embryonic fibroblasts (MEFs) by promoting p53‐mediated transactivation.^38^ However, the recruitment of H3K18ac and H3K27ac into COL1a1 promoter is increased in a high glucose–induced renal fibrosis model by MRTF‐A and p300.^39^ Our data demonstrated that increased acetylation of H3K18 and H3K27 resulted in decreased collagen expression, indicating a different transcription of acetylation of H3K18ac and H3K27 in Ang II–treated CFs. According to the concomitant changes in p53, we speculate that the role of acetylation of H3K18 and H3K27 on cell proliferation and fibrotic gene expression depends on the status of p53 during Ang II–induced cardiac fibrosis.

In summary, this report identifies PI16 as an anti‐fibrosis regulator of CFs by modulating HDAC1 and p53 expression. Targeting an endogenous HDAC1 inhibitor may offer a more specific treatment option with less adverse effects for patients with cardiac fibrosis.

## CONFLICTS OF INTEREST

None.

## AUTHOR CONTRIBUTIONS

WS and XK conceived and designed experiments and interpreted data. MD, SY, YL, MQ and YJ performed experiments. WS, MD and XK analysed the data and compiled the figures. MD, WS and XK wrote and edited the manuscript.

## Supporting information

Supplementary Material

## Data Availability

All data are available in the manuscript or the Supporting Information.
