# Supplementary material for: Overexpression of peptidase inhibitor 16 attenuates angiotensin II–induced cardiac fibrosis via regulating HDAC1 of cardiac fibroblasts
Source: J Cell Mol Med. 2020 Mar 30;24(9):5249–59. doi: 10.1111/jcmm.15178 (PMC7205788; doi:10.1111/jcmm.15178)
Supplement: Supplementary file 1 — Supplementary Material [file JCMM-24-5249-s001.docx]

**Supplemental Materials**

**Supplemental Table 1**

Echocardiographic assessment of cardiac function in WT and PI16-Tg mice infused with a pressor dose of Ang II (3.6mg/kg/d). IVS;d = end-diastolic interventricular septal thickness, IVS;s = end-systolic interventricular septal thickness, LVID;d = end-diastolic left ventricular internal diameter, LVID;s = end-systolic left ventricular internal diameter, LVPW;d = end-diastolic left ventricular posterior wall thickness, LVPW;s = end-systolic left ventricular posterior wall thickness, LV = left ventricle, EF = ejection fraction, HR = heart rate. Results are presented as mean ± S.E.M. *p<0.05 compared with corresponding vehicle group; #p<0.05 compared with WT + Ang II group.

|  | WT Sham | WT AngII | PI16-Tg Sham | PI16-Tg AngII |
| --- | --- | --- | --- | --- |
| n | 9 | 11 | 9 | 11 |
| IVS;d(mm) | 0.71±0.03 | 1.01±0.10* | 0.68±0.05 | 1.00±0.13* |
| IVS;s(mm) | 1.09±0.04 | 1.38±0.13* | 1.08±0.08 | 1.33±0.19* |
| LVID;d(mm) | 3.44±0.15 | 3.62±0.41 | 3.39±0.20 | 3.23±0.32# |
| LVID;s(mm) | 2.26±0.12 | 2.48±0.44 | 2.18±0.19 | 2.27±0.40 |
| LVPW;d(mm) | 0.71±0.03 | 0.98±0.12* | 0.69±0.04 | 0.98±0.15* |
| LVPW;s(mm) | 1.15±0.07 | 1.38±0.19* | 1.14±0.08 | 1.30±0.21 |
| LV EF(%) | 64.70±1.56 | 46.62±4.05 | 66.25±3.30 | 57.96±9.72# |
| LV mass(mg) | 78.16±5.37 | 135.38±16.84* | 72.29±7.89 | 113.18±18.78*,# |
| HR(bpm) | 487.53±39.68 | 510.53±36.88 | 484.93±41.57 | 507.80±35.66 |

**
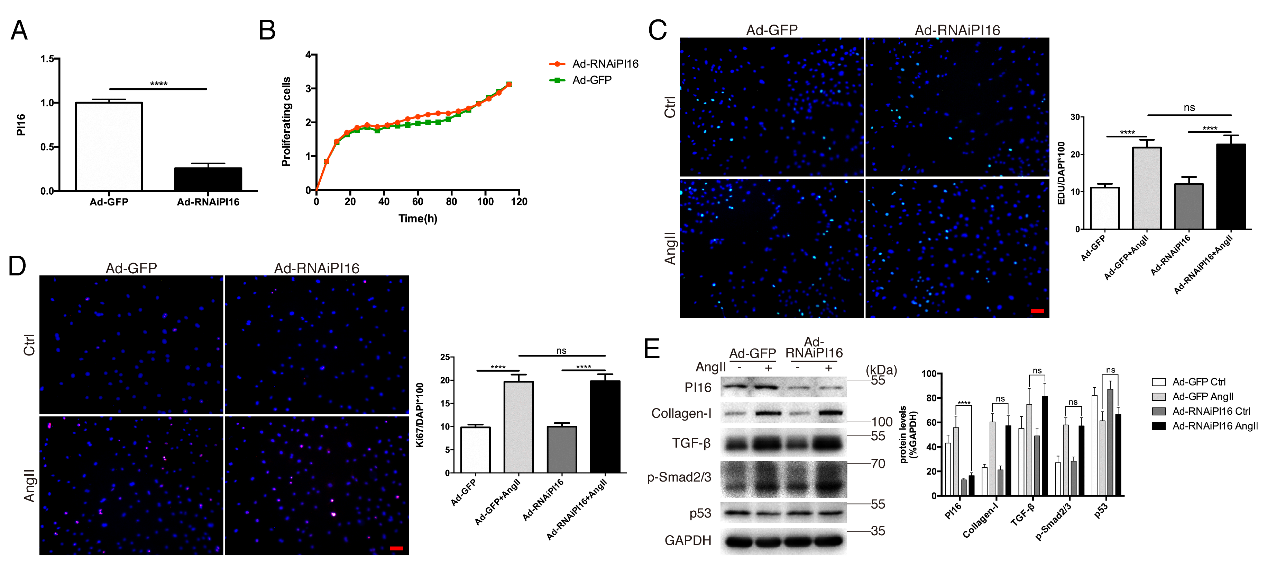
**

**Supplemental Figure 1. Knockdown of PI16 does not affect NRCF proliferation.** (A) mRNA levels of PI16 in NRCFs were assessed by qPCR. (B) Cell proliferation assessed by RTCA using electric impedance as a measure of NRCFs transfected with Ad-RNAiPI16 or Ad-GFP proliferation every 15 min. Electrical impedance was normalized according to the background measurement at time point 0. (C) Representative images and quantitation of Edu-labeled (green) NRCFs with Ad-RNAiPI16 or Ad-GFP transfection and Ang II treatment. Scale bar, 50 μm. (D) Representative images and quantitation of Ki67-labeled (red) NRCFs with Ad-RNAiPI16 or Ad-GFP transfection and Ang II treatment. Scale bar, 50 μm. (E) Protein levels of PI16, collagen I, TGF-β, p-Smad2/3, and p53 in NRCFs were assessed by western blotting. The relative protein levels were normalized to GAPDH. Data are shown as the means ± standard errors of the mean of triplicates and are representative of three independent experiments performed. ns=none sense. ****p < 0.0001.

**
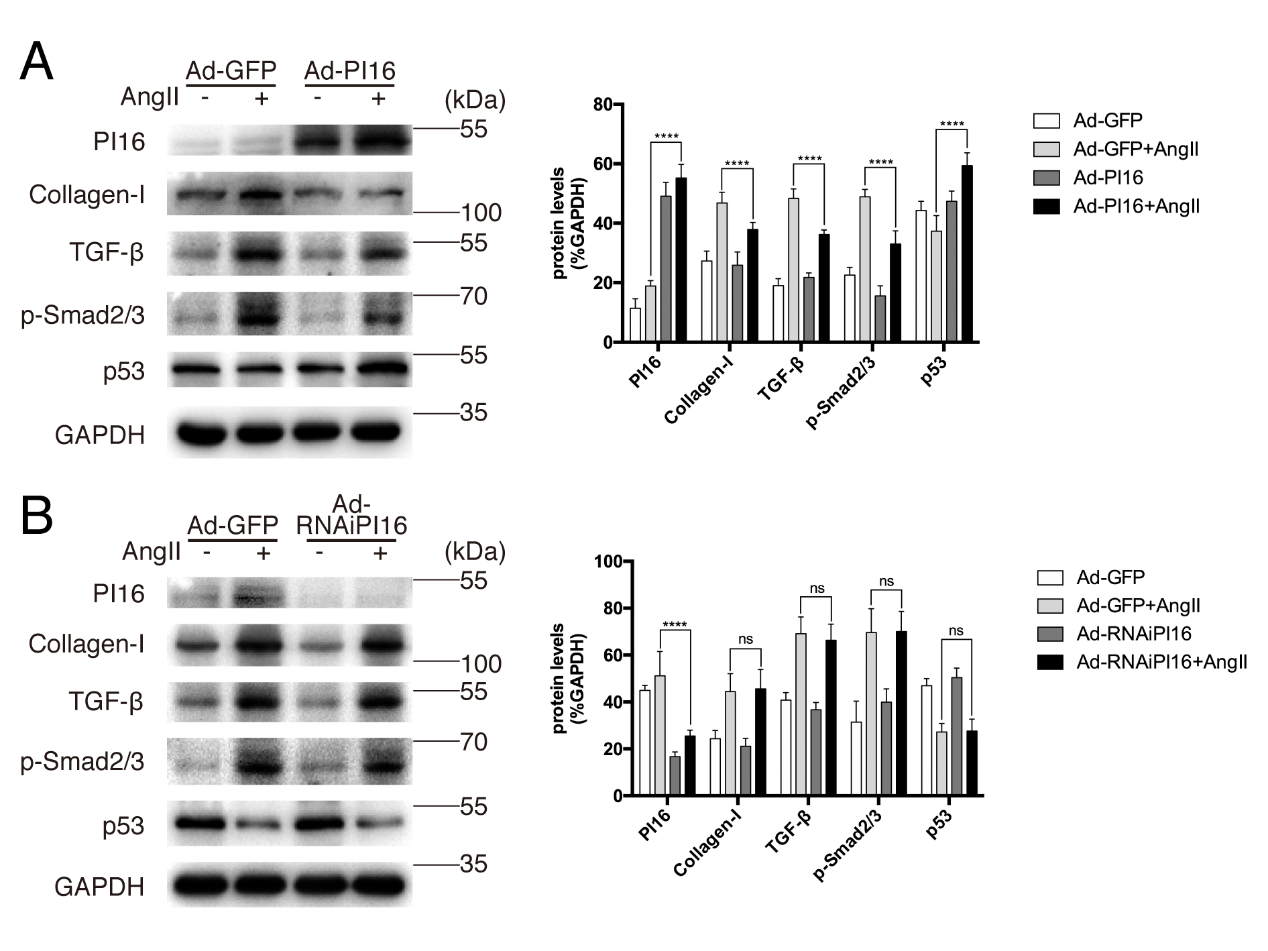
**

Supplemental Figure 2. Overexpression of PI16 attenuates angiotensin II (Ang II)–induced fibrotic gene expression in adult rat cardiac fibroblasts while knockdown of PI16 does not. (A) Protein levels of PI16, collagen I, TGF-β, p-Smad2/3, and p53 in adult rat cardiac fibroblasts (ARCFs) with Ad-PI16 or Ad-GFP transfection and AngII treatment were assessed by western blotting. The relative protein levels were normalized to GAPDH. (B) Protein levels of PI16, collagen I, TGF-β, p-Smad2/3, and p53 in adult rat cardiac fibroblasts (ARCFs) with PI16 knockdown and AngII treatment were assessed by western blotting. The relative protein levels were normalized to GAPDH. Data are shown as the means ± standard errors of the mean of triplicates and are representative of three independent experiments performed. ns=none sense. ****p < 0.0001.


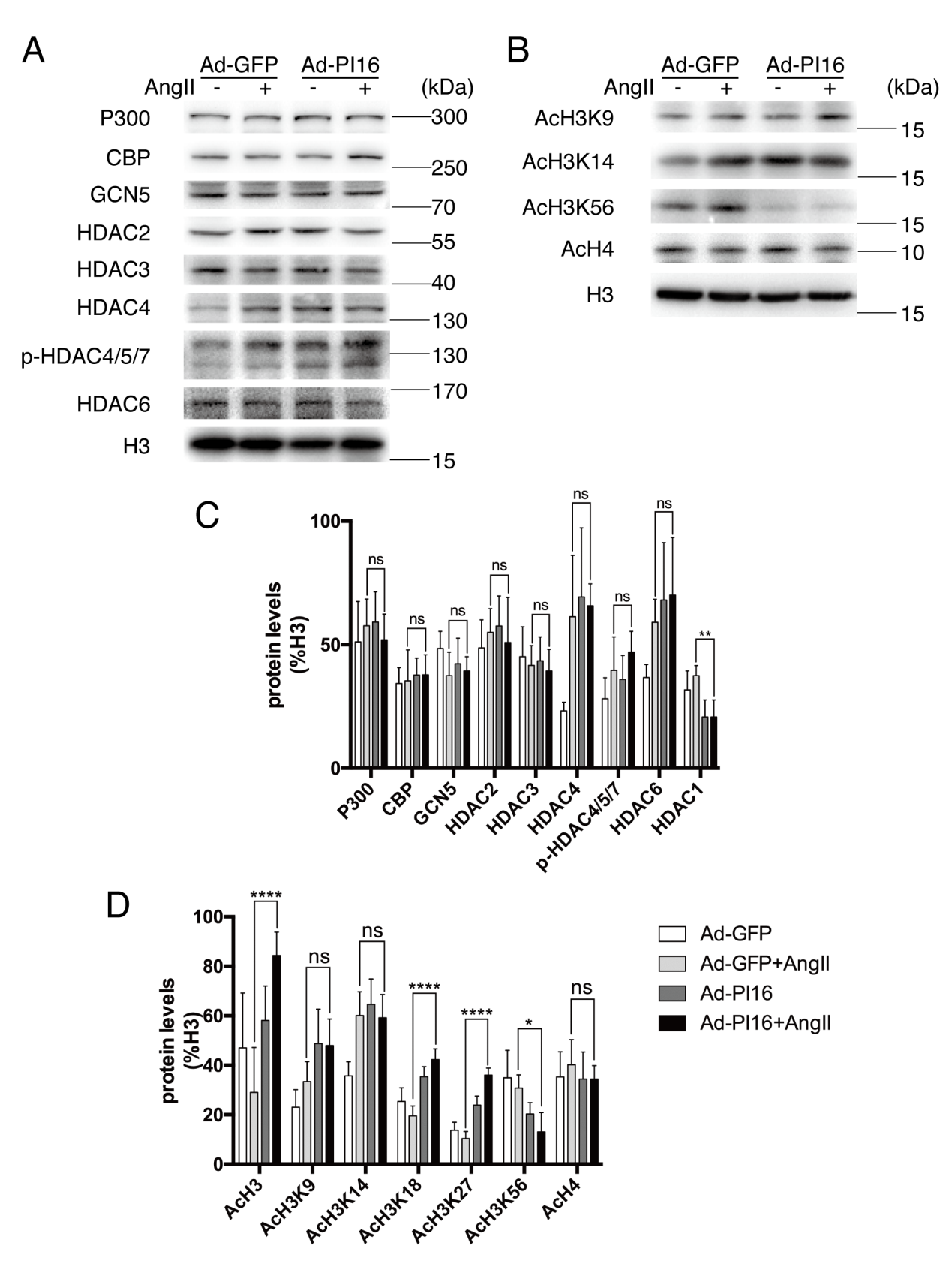


**Supplemental Figure 3. Protein levels of histone acetyltransferase and histone deacetylases, and modification of H3 and H4 in NRCFs.** (A, C) Nuclear protein levels of P300, CBP, GCN5, HDAC2, HDAC3, HDAC4, p-HDAC4/5/7, and HDAC6 in NRCFs were assessed by western blotting. The relative protein levels were normalized to histone 3 (H3). (B, D) Nuclear protein levels of AcH3k9, AcH3K14, AcH3K56, and AcH4 were assessed by western blotting. The relative protein levels were normalized to H3. Data are shown as the means ± standard errors of the mean of triplicates and are representative of three independent experiments performed. ns=none sense. *p < 0.05, **p < 0.01, ****p < 0.0001.

**
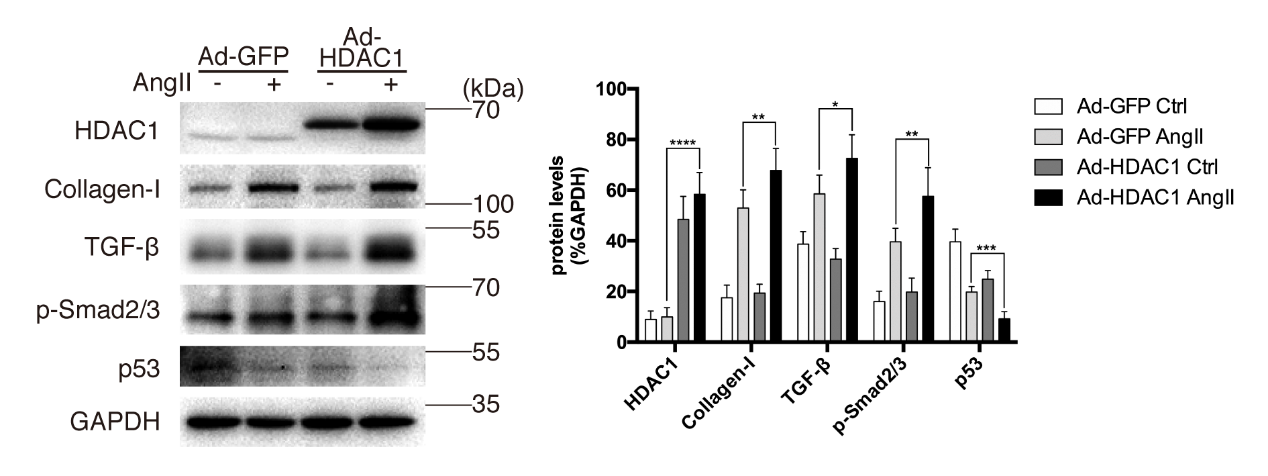
**

**Supplemental Figure 4. Overexpression of HDAC1 promotes fibrotic gene expression in NRCFs.** Protein levels of HDAC1, collagen I, TGF-β, p-Smad2/3, and p53 in NRCFs were assessed by western blotting. The relative protein levels were normalized to GAPDH. Data are shown as the means ± standard errors of the mean of triplicates and are representative of three independent experiments performed. *p < 0.05, **p < 0.01, ***p < 0.001, ****p < 0.0001.

**
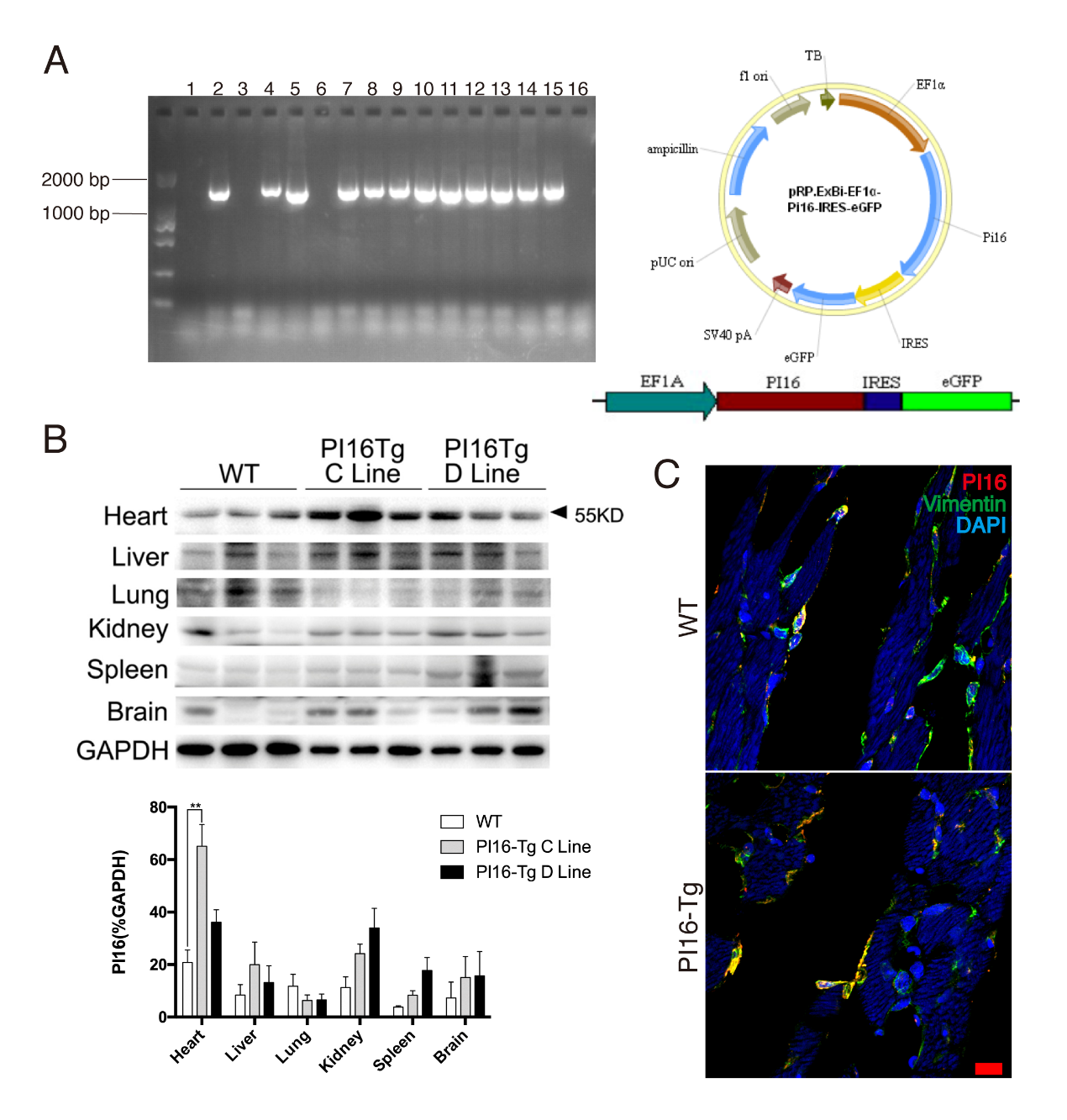
**

**Supplemental Figure 5. Generation of PI16 transgenic mice.** (A) Representative image of PCR genotyping for PI16 (1550 bp). Lane 2, 4, 5 and 7-15 were genotyped to be PI16-Tg positive mice. PI16 transgenic mice were generated using the EF1-α promoter system. Structure of expression vector pRP.EX3d -EF1A >Pi16 >IRES/eGFP was illustrated. (B) Protein levels of PI16 in different tissues from wild-type (WT) and two lines of PI16 transgenic (PI16-Tg) mice. PI16 was specifically high-expressed in the hearts of PI16-Tg C line mice. (C) Immunofluorescent staining of PI16 in hearts from wild type or PI16-Tg mice. To confirm the co-localization of PI16 and vimentin, individual channels and merged images are all shown. Scale bar, 20 μm. **p < 0.01.


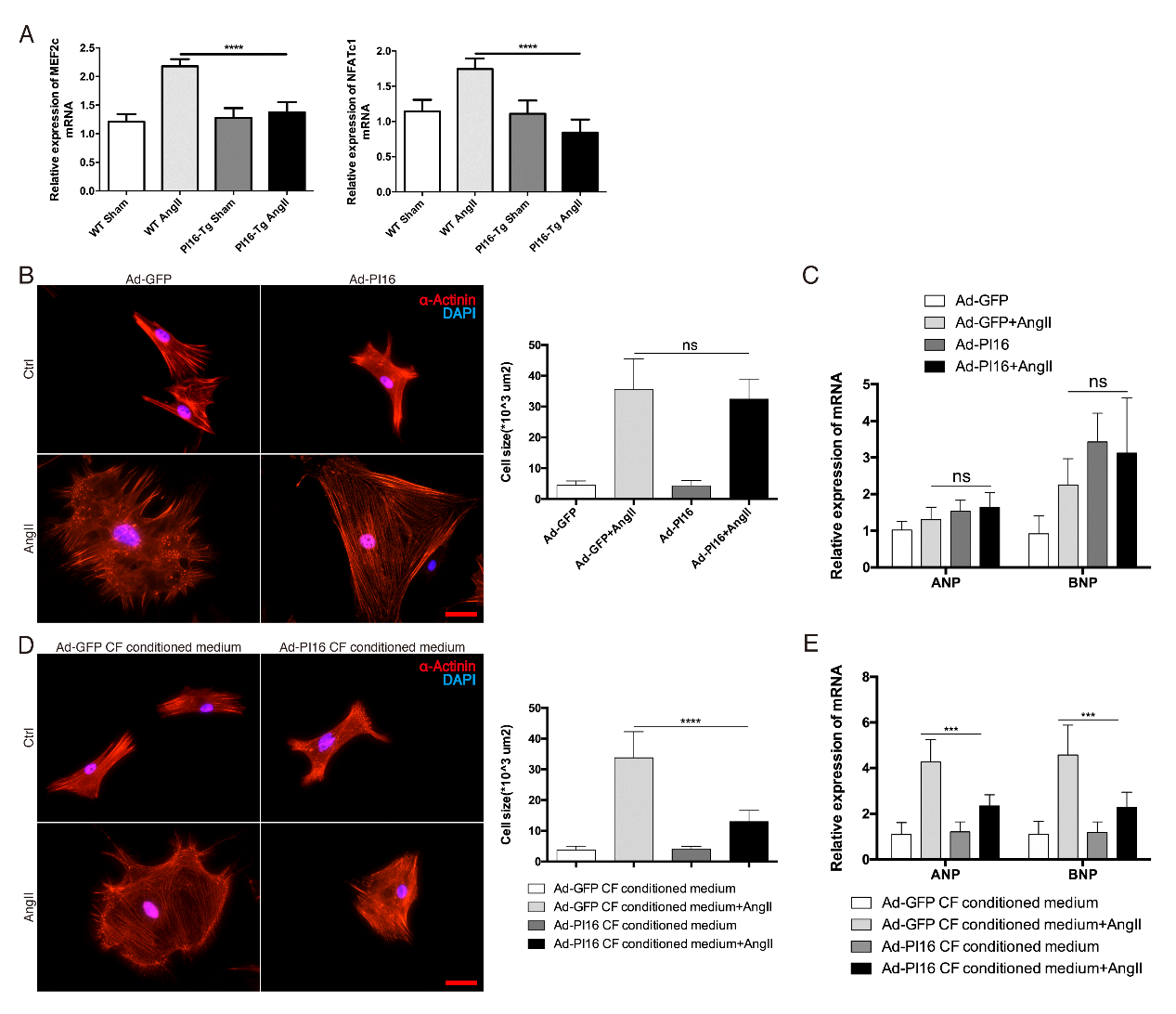


**Supplemental Figure 6. Overexpression of PI16 attenuates angiotensin II–induced cardiomyocyte hypertrophy in vivo.** (A) mRNA levels of MEF2c and NFATc1 in hearts were assessed by qPCR. (B) Representative images of immunofluorescent staining and quantitation of cell size of NRVMs (red) with Ad-PI16 or Ad-GFP transfection and Ang II treatment. Scale bar, 50 μm. Data showed that overexpression of PI16 in newborn rat ventricular myocytes (NRVMs) does not attenuate angiotensin II–induced cardiomyocyte hypertrophy. (C) mRNA levels of ANP and BNP in NRVMs with Ad-PI16 or Ad-GFP transfection and Ang II treatment were assessed by qPCR. (D) Representative images of immunofluorescent staining and quantitation of cell size of NRVMs (red) treated with NRCFs conditioned medium and AngII. Scale bar, 50 μm. Data showed that PI16 expressed by CFs has paracrine effects on attenuating angiotensin II–induced cardiomyocyte hypertrophy. (E) mRNA levels of ANP and BNP in NRVMs treated with NRCFs conditioned medium and AngII were assessed by qPCR. Data are shown as the means ± standard errors of the mean of triplicates and are representative of three independent experiments performed. ns=none sense. ***p < 0.001, ****p < 0.0001.
